# Supplementary material for: Pyruvate Dehydrogenase Kinase 1 inhibition mediated oxidative phosphorylation enhancement in cartilage promotes osteoarthritis progression
Source: BMC Musculoskelet Disord. 2023 Jul 20;24:597. doi: 10.1186/s12891-023-06585-6 (PMC10357736; doi:10.1186/s12891-023-06585-6)
Supplement: Supplementary file 1 — Additional file 1. [file 12891_2023_6585_MOESM1_ESM.zip › KEGG.Gsea.1653622432464/KEGG_PPAR_SIGNALING_PATHWAY.html]

Details for gene set KEGG\_PPAR\_SIGNALING\_PATHWAY[GSEA]

|  || Dataset | 55457\_collapsed\_to\_symbols.cls.cls #OA\_versus\_normal.cls.cls #OA\_versus\_normal\_repos |
| Phenotype | cls.cls#OA\_versus\_normal\_repos |
| Upregulated in class | normal |
| GeneSet | KEGG\_PPAR\_SIGNALING\_PATHWAY |
| Enrichment Score (ES) | -0.15821648 |
| Normalized Enrichment Score (NES) | -0.6304374 |
| Nominal p-value | 0.9920382 |
| FDR q-value | 1.0 |
| FWER p-Value | 1.0 |
Table: GSEA Results Summary

  

Fig 1: Enrichment plot: KEGG\_PPAR\_SIGNALING\_PATHWAY      
 Profile of the Running ES Score & Positions of GeneSet Members on the Rank Ordered List

  

| SYMBOL | TITLE | RANK IN GENE LIST | RANK METRIC SCORE | RUNNING ES | CORE ENRICHMENT || 1 | SCP2 | sterol carrier protein 2 [Source:HGNC Symbol;Acc:HGNC:10606] | 105 | 0.905 | 0.0436 | Yes |
| 2 | CYP27A1 | cytochrome P450 family 27 subfamily A member 1 [Source:HGNC Symbol;Acc:HGNC:2605] | 611 | 0.619 | 0.0398 | Yes |
| 3 | PCK2 | "phosphoenolpyruvate carboxykinase 2, mitochondrial [Source:HGNC Symbol;Acc:HGNC:8725]" | 759 | 0.579 | 0.0615 | Yes |
| 4 | OLR1 | oxidized low density lipoprotein receptor 1 [Source:HGNC Symbol;Acc:HGNC:8133] | 1077 | 0.500 | 0.0654 | Yes |
| 5 | SLC27A6 | solute carrier family 27 member 6 [Source:HGNC Symbol;Acc:HGNC:11000] | 1182 | 0.480 | 0.0848 | Yes |
| 6 | ILK | integrin linked kinase [Source:HGNC Symbol;Acc:HGNC:6040] | 1286 | 0.460 | 0.1032 | Yes |
| 7 | ACOX1 | acyl-CoA oxidase 1 [Source:HGNC Symbol;Acc:HGNC:119] | 1401 | 0.443 | 0.1196 | Yes |
| 8 | NR1H3 | nuclear receptor subfamily 1 group H member 3 [Source:HGNC Symbol;Acc:HGNC:7966] | 1728 | 0.394 | 0.1168 | Yes |
| 9 | ME1 | malic enzyme 1 [Source:HGNC Symbol;Acc:HGNC:6983] | 1746 | 0.392 | 0.1379 | Yes |
| 10 | HMGCS2 | 3-hydroxy-3-methylglutaryl-CoA synthase 2 [Source:HGNC Symbol;Acc:HGNC:5008] | 1888 | 0.374 | 0.1484 | Yes |
| 11 | UCP1 | uncoupling protein 1 [Source:HGNC Symbol;Acc:HGNC:12517] | 2339 | 0.322 | 0.1318 | No |
| 12 | GK2 | glycerol kinase 2 [Source:HGNC Symbol;Acc:HGNC:4291] | 3042 | 0.246 | 0.0912 | No |
| 13 | ACOX2 | acyl-CoA oxidase 2 [Source:HGNC Symbol;Acc:HGNC:120] | 3176 | 0.233 | 0.0941 | No |
| 14 | FABP6 | fatty acid binding protein 6 [Source:HGNC Symbol;Acc:HGNC:3561] | 3312 | 0.220 | 0.0962 | No |
| 15 | SLC27A5 | solute carrier family 27 member 5 [Source:HGNC Symbol;Acc:HGNC:10999] | 3525 | 0.200 | 0.0911 | No |
| 16 | MMP1 | matrix metallopeptidase 1 [Source:HGNC Symbol;Acc:HGNC:7155] | 3625 | 0.191 | 0.0944 | No |
| 17 | CPT2 | carnitine palmitoyltransferase 2 [Source:HGNC Symbol;Acc:HGNC:2330] | 3742 | 0.182 | 0.0958 | No |
| 18 | CYP7A1 | cytochrome P450 family 7 subfamily A member 1 [Source:HGNC Symbol;Acc:HGNC:2651] | 4499 | 0.113 | 0.0434 | No |
| 19 | ACADM | acyl-CoA dehydrogenase medium chain [Source:HGNC Symbol;Acc:HGNC:89] | 4983 | 0.074 | 0.0100 | No |
| 20 | FADS2 | fatty acid desaturase 2 [Source:HGNC Symbol;Acc:HGNC:3575] | 5025 | 0.071 | 0.0108 | No |
| 21 | SLC27A2 | solute carrier family 27 member 2 [Source:HGNC Symbol;Acc:HGNC:10996] | 5122 | 0.062 | 0.0069 | No |
| 22 | ACAA1 | acetyl-CoA acyltransferase 1 [Source:HGNC Symbol;Acc:HGNC:82] | 5175 | 0.058 | 0.0062 | No |
| 23 | FABP3 | fatty acid binding protein 3 [Source:HGNC Symbol;Acc:HGNC:3557] | 5818 | 0.008 | -0.0434 | No |
| 24 | PPARG | peroxisome proliferator activated receptor gamma [Source:HGNC Symbol;Acc:HGNC:9236] | 6006 | -0.005 | -0.0577 | No |
| 25 | FABP7 | fatty acid binding protein 7 [Source:HGNC Symbol;Acc:HGNC:3562] | 6387 | -0.034 | -0.0853 | No |
| 26 | ACADL | acyl-CoA dehydrogenase long chain [Source:HGNC Symbol;Acc:HGNC:88] | 6427 | -0.037 | -0.0862 | No |
| 27 | ACSL6 | acyl-CoA synthetase long chain family member 6 [Source:HGNC Symbol;Acc:HGNC:16496] | 6505 | -0.043 | -0.0898 | No |
| 28 | ACOX3 | "acyl-CoA oxidase 3, pristanoyl [Source:HGNC Symbol;Acc:HGNC:121]" | 6747 | -0.059 | -0.1052 | No |
| 29 | RXRG | retinoid X receptor gamma [Source:HGNC Symbol;Acc:HGNC:10479] | 6947 | -0.075 | -0.1163 | No |
| 30 | RXRA | retinoid X receptor alpha [Source:HGNC Symbol;Acc:HGNC:10477] | 7381 | -0.109 | -0.1438 | No |
| 31 | PLIN1 | perilipin 1 [Source:HGNC Symbol;Acc:HGNC:9076] | 7402 | -0.111 | -0.1391 | No |
| 32 | PLTP | phospholipid transfer protein [Source:HGNC Symbol;Acc:HGNC:9093] | 7412 | -0.112 | -0.1334 | No |
| 33 | FABP1 | fatty acid binding protein 1 [Source:HGNC Symbol;Acc:HGNC:3555] | 7604 | -0.127 | -0.1409 | No |
| 34 | FABP5 | fatty acid binding protein 5 [Source:HGNC Symbol;Acc:HGNC:3560] | 7692 | -0.134 | -0.1401 | No |
| 35 | FABP4 | fatty acid binding protein 4 [Source:HGNC Symbol;Acc:HGNC:3559] | 7741 | -0.137 | -0.1359 | No |
| 36 | ADIPOQ | "adiponectin, C1Q and collagen domain containing [Source:HGNC Symbol;Acc:HGNC:13633]" | 7792 | -0.141 | -0.1318 | No |
| 37 | DBI | "diazepam binding inhibitor, acyl-CoA binding protein [Source:HGNC Symbol;Acc:HGNC:2690]" | 7929 | -0.149 | -0.1338 | No |
| 38 | EHHADH | enoyl-CoA hydratase and 3-hydroxyacyl CoA dehydrogenase [Source:HGNC Symbol;Acc:HGNC:3247] | 7968 | -0.153 | -0.1280 | No |
| 39 | CD36 | CD36 molecule [Source:HGNC Symbol;Acc:HGNC:1663] | 7985 | -0.154 | -0.1205 | No |
| 40 | CPT1A | carnitine palmitoyltransferase 1A [Source:HGNC Symbol;Acc:HGNC:2328] | 8046 | -0.159 | -0.1161 | No |
| 41 | AQP7 | aquaporin 7 [Source:HGNC Symbol;Acc:HGNC:640] | 8136 | -0.165 | -0.1135 | No |
| 42 | ACSL3 | acyl-CoA synthetase long chain family member 3 [Source:HGNC Symbol;Acc:HGNC:3570] | 8170 | -0.169 | -0.1064 | No |
| 43 | LPL | lipoprotein lipase [Source:HGNC Symbol;Acc:HGNC:6677] | 8374 | -0.184 | -0.1117 | No |
| 44 | SORBS1 | sorbin and SH3 domain containing 1 [Source:HGNC Symbol;Acc:HGNC:14565] | 8509 | -0.194 | -0.1110 | No |
| 45 | CYP4A11 | cytochrome P450 family 4 subfamily A member 11 [Source:HGNC Symbol;Acc:HGNC:2642] | 8654 | -0.205 | -0.1105 | No |
| 46 | APOA1 | apolipoprotein A1 [Source:HGNC Symbol;Acc:HGNC:600] | 8882 | -0.223 | -0.1154 | No |
| 47 | APOC3 | apolipoprotein C3 [Source:HGNC Symbol;Acc:HGNC:610] | 8901 | -0.225 | -0.1040 | No |
| 48 | PCK1 | phosphoenolpyruvate carboxykinase 1 [Source:HGNC Symbol;Acc:HGNC:8724] | 9100 | -0.239 | -0.1057 | No |
| 49 | ACSL5 | acyl-CoA synthetase long chain family member 5 [Source:HGNC Symbol;Acc:HGNC:16526] | 9136 | -0.243 | -0.0945 | No |
| 50 | SCD | stearoyl-CoA desaturase [Source:HGNC Symbol;Acc:HGNC:10571] | 9824 | -0.303 | -0.1306 | No |
| 51 | CPT1B | carnitine palmitoyltransferase 1B [Source:HGNC Symbol;Acc:HGNC:2329] | 10179 | -0.339 | -0.1388 | No |
| 52 | GK | glycerol kinase [Source:HGNC Symbol;Acc:HGNC:4289] | 10291 | -0.350 | -0.1274 | No |
| 53 | FABP2 | fatty acid binding protein 2 [Source:HGNC Symbol;Acc:HGNC:3556] | 10547 | -0.378 | -0.1257 | No |
| 54 | ACSL1 | acyl-CoA synthetase long chain family member 1 [Source:HGNC Symbol;Acc:HGNC:3569] | 10705 | -0.394 | -0.1153 | No |
| 55 | PPARA | peroxisome proliferator activated receptor alpha [Source:HGNC Symbol;Acc:HGNC:9232] | 11110 | -0.445 | -0.1213 | No |
| 56 | PPARD | peroxisome proliferator activated receptor delta [Source:HGNC Symbol;Acc:HGNC:9235] | 11301 | -0.471 | -0.1091 | No |
| 57 | ACSL4 | acyl-CoA synthetase long chain family member 4 [Source:HGNC Symbol;Acc:HGNC:3571] | 11327 | -0.475 | -0.0839 | No |
| 58 | APOA2 | apolipoprotein A2 [Source:HGNC Symbol;Acc:HGNC:601] | 11383 | -0.483 | -0.0605 | No |
| 59 | ANGPTL4 | angiopoietin like 4 [Source:HGNC Symbol;Acc:HGNC:16039] | 11389 | -0.484 | -0.0332 | No |
| 60 | PDPK1 | 3-phosphoinositide dependent protein kinase 1 [Source:HGNC Symbol;Acc:HGNC:8816] | 11839 | -0.557 | -0.0362 | No |
| 61 | RXRB | retinoid X receptor beta [Source:HGNC Symbol;Acc:HGNC:10478] | 11871 | -0.565 | -0.0063 | No |
| 62 | UBC | ubiquitin C [Source:HGNC Symbol;Acc:HGNC:12468] | 12411 | -0.710 | -0.0076 | No |
| 63 | SCD5 | stearoyl-CoA desaturase 5 [Source:HGNC Symbol;Acc:HGNC:21088] | 12597 | -0.796 | 0.0236 | No |
Table: GSEA details [plain text format]

  

Fig 2: KEGG\_PPAR\_SIGNALING\_PATHWAY      
 Blue-Pink O' Gram in the Space of the Analyzed GeneSet

  

Fig 3: KEGG\_PPAR\_SIGNALING\_PATHWAY: Random ES distribution      
 Gene set null distribution of ES for **KEGG\_PPAR\_SIGNALING\_PATHWAY**
